# Early admission is better–the time to admission (TTA) is associated with one-year mortality in hip fracture

**DOI:** 10.1097/JS9.0000000000001835

**Published:** 2024-06-17

**Authors:** Bin-Fei Zhang, Shuai-Liang Xu, Zhi Yang, Peng Xu

**Affiliations:** Department of Joint Surgery, Honghui Hospital, Xi’an Jiaotong University, Xi’an, Shaanxi Province, People’s Republic of China

**Keywords:** complication, hip fracture, one-year mortality, TTA

## Abstract

**Objective:**

This study evaluated the probable association between time to admission (TTA) and 1-year mortality in geriatric hip fractures.

**Methods:**

Older adult patients with hip fractures were screened between January 2015 and September 2019. The demographic and clinical characteristics of the patients were collected at the largest trauma center in Northwest China. TTA can be obtained from the medical record system and converted into a categorical variable. Multivariate binary logistic regression and generalized additive model were used to identify the linear and nonlinear association between TTA and 1-year mortality. Analyses were performed using EmpowerStats and the R software.

**Results:**

Two thousand three hundred and sixty-one patients who met the criteria were finally included. There were 1618 (68.53%) female and 743 (31.47%) male patients. All patients were divided into three groups according to their TTA. The proportions of patients with low (≤6 h), middle (>6, ≤24 h), and high (>24 h) waiting times were 995, 654, and 712, respectively, and the corresponding 1-year mortality rates were 62 (6.23%), 72 (11.01%), and 82 (11.52%). We found a curve relationship between TTA and 1-year mortality by two-piecewise linear regression, and 9 h was an inflection point. When TTA was less than 9 h, the 1-year mortality of patients increased by 9% for every 1 h increase in TTA (OR=1.09, 95% CI: 1.03–1.16; *P*<0.01). When TTA was greater than 9 h, the mortality of patients no longer increased with the rise of TTA (OR=1.00, 95% CI: 1.00–1.00; *P*=0.26).

**Conclusion:**

TTA is a probable predictor of 1-year mortality. The authors found that 9 h was an inflection point. If TTA is less than 9 h, the mortality rate of patients will be lower. If it takes more than 9 h, the mortality will be higher. Therefore, the elderly who are found to have possible hip fractures should be admitted to the hospital as soon as possible.

## Introduction

Highlights

- We found a curve relationship between time to admission (TTA) and 1-year mortality by two-piecewise linear regression, and 9 h was an inflection point.
- When TTA was less than 9 h, the 1-year mortality increased by 9% for every 1 h increase in TTA. When TTA was greater than 9 h, the mortality of patients no longer increased with the rise of TTA.
- The elderly with possible hip fractures should be admitted to the hospital as soon as possible.

A hip fracture is a severe osteoporotic fracture. In older people, the common mechanism of hip fracture is low-energy trauma caused by falls. The incidence of hip fracture over 65 years was 278/100 000 in China between 2013 and 2016^1^. About 2 million older adults worldwide suffer from hip fractures yearly^2,3^. With the ageing of the global population, the number of patients will continue to rise. By 2050, hip fractures are expected to reach 4.5–6.3 million^2,4^.

Hip fracture is a significant cause of mortality in the elderly population worldwide. Previous studies have shown that 1-year mortality after hip fracture is 20–30%^1–3^. Meanwhile, the investigators estimated that patients with hip fractures have excess annual mortality than those without fractures by 20–22% 10 years after injury^5^. In addition, the disability rate of hip fractures is as high as 50%, which leads to a decline in the quality of life of patients and a substantial psychological and economic burden. The early operation has been proved to be an effective strategy for hip fractures^6^. It has the advantages of good joint activity, early ambulation, and reducing the complications of long-term bed rest in elderly patients.

To improve the prognosis of hip fractures, scholars have researched many related factors, one of which is the relationship between preoperative waiting time and postoperative mortality and complications. Generally, the so-called preoperative waiting time should be the time from injury to receiving the surgery. Most studies have revealed the association between postadmission waiting time (hours from admission to start of surgery) and postoperative mortality and complication^7,8^. The guidelines in different countries suggest the corresponding operation time is 36 or 48 h after admission^9,10^. However, due to the inhomogeneous medical resources in various countries and regions, most studies only consider the impact of the time from admission to surgery on the prognosis while ignoring the delay from injury time to admission (TTA)^11,12^. Therefore, the primary purpose of this study is to explore the probable association between TTA and prognosis in geriatric hip fractures, which will help surgeons ensure surgical safety, reduce mortality, help individualized risk assessment, and prevent adverse outcomes.

## Materials and methods

### Study design

In this retrospective cohort study, we recruited older adults who had a hip fracture from 1 January 2015 to 30 September 2019 at the largest trauma center in Northwest China.

This retrospective study was approved by the Ethics Committee of our Hospital (No. 202201009). All patients signed related informed consent before the operation. All human-related procedures followed the 1964 Declaration of Helsinki and its later amendments. The study has been reported according to the strengthening the reporting of cohort, cross-sectional, and case–control studies in surgery (STROCSS) 2021 guidelines^13^.

### Participants

Demographic and clinical data of the patients were obtained from their original medical records. The inclusion criteria were as follows: (1) Age ≥65 years; (2) X-ray or computed tomography diagnosis of the femoral neck or intertrochanteric fracture; (3) patients who were receiving surgical treatment in the hospital; (4) availability of clinical data in the hospital.

### Hospital treatment

All patients were diagnosed with hip fractures by X-ray examination at admission. The hospital treatment of hip fractures is divided into three types according to the patient’s age and personal condition: closed/open reduction internal fixation (CRIF/ORIF), hemiarthroplasty (HA), and total hip arthroplasty (THA). For patients with intertrochanteric fractures, CRIF/ORIF was usually performed. The patient was placed on a traction table, and then a proximal femoral nail antirotation was inserted by minimal incision after getting a good reduction. For patients with femoral neck fractures, arthroplasty is usually performed. We used the posterior approach to undergo the femoral head or total hip arthroplasty procedure. The senior surgeons mainly performed all operations, and other surgeons were surgeon’s assistants. The patients receiving CRIF/ORIF and receiving arthroplasty were asked to weight bearing when 2 weeks and 2 days after the operations, respectively.

### Endpoint events

The endpoint event in this study was the 1-year mortality rate after surgery.

### Variables

The variables collected in this study were as follows^14^: demographic variables (age, sex, injury mechanism, fracture classification, hypertension, diabetes, arrhythmia, hemorrhagic stroke, dementia, ischemic stroke, cancer, multiple injuries, chronic obstructive pulmonary disease, hepatitis, gastritis, and aCCI (age-adjusted Charlson Comorbidity Index), clinical variables (treatment strategy, TTA, operation time, blood loss, infusion, and stay in hospital). The dependent variable was 1-year mortality, and the independent variable was the TTA. Other variables were potentially confounding factors.

### Follow-up

After discharge, patients’ family members were contacted by telephone from January 2022 to March 2022 to record survival and survival time data. Telephone follow-up was conducted by two medical professionals.

### Statistics analysis

Continuous variables are reported as mean±SD or median (min, max), and categorical variables are given as frequencies and percentages. We used *χ* ^2^, the One-way ANOVA test, or the Kruskal–Wallis *H* test for differences among different TTA subgroups (tertiles). Based on the criteria of *P*<0.1 in univariate analysis and the previously reported factors, we selected the variables and introduced them into multivariate analysis. We used the multivariate binary logistic regression model to test the TTA and 1-year mortality association. To test the robustness of our results, we performed a sensitivity analysis. We converted TTA into a categorical variable according to the tertiles. We calculated the *P* for the trend to verify the results of TTA as the continuous variable and to examine the possibility of nonlinearity.

To account for the nonlinear relationship between TTA and 1-year mortality, we used a generalized additive model and the smooth curve fitting (penalized spline method) to address nonlinearity. Besides, the two-piecewise binary logistic regression model was also used to explain the nonlinearity further. In addition, we draw the Kaplan–Meier survival curves to illustrate the trend of in different TTA subgroups.

All analyses were performed using statistical software packages R (http://www.R-project.org, R Foundation) and EmpowerStats (http://www.empowerstats.com, X&Y Solutions Inc.). Odds ratios (OR) and 95% CI were calculated. A *P*-value <0.05 (two-sided) was considered to represent statistical significance.

## Results

### Patient characteristics

**Figure 1:**
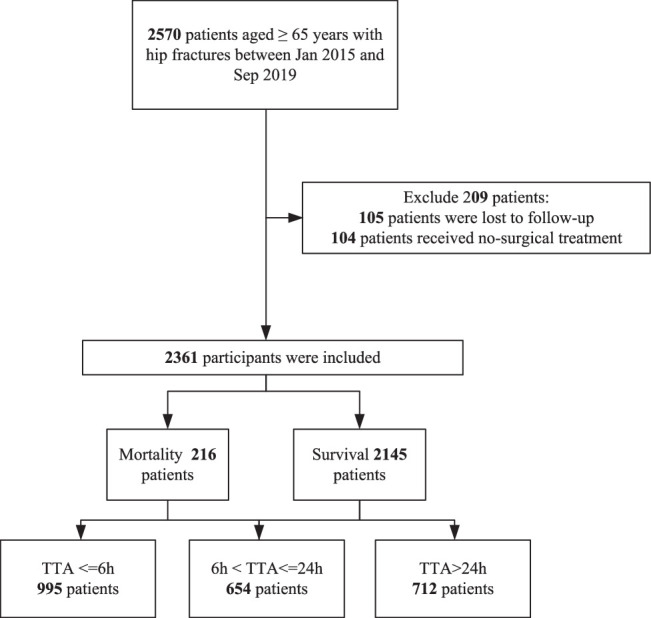
The flow chart in the process of including patients.

A total of 2361 patients who met the inclusion criteria were included in our study. The flow chart is shown in Figure 1. We divided the patients into three groups according to TTA. The general information of patients is shown in Table 1. There were 743 males and 1618 females, with a mean age of 79.3±6.7 years. The injury mechanism included 2286 (96.8%) falls, 61 (2.6%) accidents, and 14 (0.6%) other reasons. There were 1745 intertrochanteric fractures and 616 femoral neck fractures. Multiple injuries occurred in 163 patients. Combined medical diseases were the following: 1215 CHD, 1154 hypertension, 459 diabetes, 48 hemorrhagic stroke, and 757 arrhythmia. There were three treatment strategies: 1724 CRIF/ORIF, 602 HA, and 35 THA. There were 1214 (51.9%) patients transferred from other hospitals, and they came to our hospital after a radiograph or computed tomography examination identified the fracture, not after hospitalization in another hospital. In addition, 1870 (80.0%) patients were injured in the daytime and 468 (20.0%) in the night-time.

**Table 1:** The demographic and clinical characteristics according to TTA tertiles.

TTA tertiles	Low (≤6 h)	Middle (>6, ≤24 h)	High ( >24 h)	*P* ^a^	*P* ^b^
*N*	995	654	712		
Age (years)	79.1±6.5	79.6±6.9	79.7±6.7	0.10	0.09
Sex				0.42	–
Male	300 (30.2%)	207 (31.7%)	236 (33.2%)		
Female	695 (69.8%)	447 (68.3%)	476 (66.8%)		
Injury mechanism				0.02	-
Low velocity trauma	961 (96.6%)	634 (96.9%)	691 (97.0%)		
High velocity trauma	30 (3.0%)	19 (2.9%)	12 (1.7%)		
Unclear	4 (0.4%)	1 (0.2%)	9 (1.3%)		
Fracture classification				<0.01	–
Fracture	789 (79.3%)	485 (74.2%)	471 (66.2%)		
Femoral neck fracture	206 (20.7%)	169 (25.8%)	241 (33.8%)		
Transferred	297 (30.0%)	268 (41.3%)	649 (92.6%)	<0.01	–
Geography					
Suburban	763 (77.2%)	459 (70.7%)	482 (68.8%)	<0.01	–
Urban	225 (22.8%)	190 (29.3%)	219 (31.2%)		
Insurance status					
Residents medical insurance	643 (65.1%)	353 (54.4%)	361 (51.5%)	<0.01	–
Employee medical insurance	345 (34.9%)	296 (45.6%)	340 (48.5%)		
Timing of injuries					
Daytime	688 (69.6%)	619 (95.4%)	563 (80.3%)	<0.01	–
Night-time	300 (30.4%)	30 (4.6%)	138 (19.7%)		
Hypertension	475 (47.7%)	300 (45.9%)	379 (53.2%)	0.02	–
Diabetes	187 (18.8%)	122 (18.7%)	150 (21.1%)	0.42	–
CHD	514 (51.7%)	337 (51.5%)	364 (51.1%)	0.98	
Arrhythmia	293 (29.5%)	206 (31.5%)	258 (36.2%)	0.01	–
Hemorrhagic stroke	11 (1.1%)	13 (2.0%)	24 (3.4%)	0.01	–
Dementia	13 (1.3%)	29 (4.4%)	47 (6.6%)	<0.01	–
Ischemic stroke	238 (23.9%)	173 (26.5%)	254 (35.7%)	<0.01	–
Cancer	28 (2.8%)	16 (2.5%)	21 (3.0%)	0.84	–
Multiple injuries	64 (6.4%)	42 (6.4%)	57 (8.0%)	0.38	–
COPD	53 (5.3%)	39 (6.0%)	45 (6.3%)	0.67	–
Hepatitis	26 (2.6%)	20 (3.1%)	24 (3.4%)	0.65	–
Gastritis	22 (2.2%)	10 (1.5%)	10 (1.4%)	0.39	–
aCCI	4.1±1.0	4.1±1.0	4.4±1.1	<0.01	<0.01
Treatment strategy				<0.01	–
CRIF/ORIF	784 (78.8%)	477 (72.9%)	463 (65.0%)		
HA	196 (19.7%)	164 (25.1%)	242 (34.0%)		
THA	15 (1.5%)	13 (2.0%)	7 (1.0%)		
TTA (h)	3.4±1.4 3 (1–6)	17.4±7.3 24 (7–24)	241.7±385.6 144 (26–5040)	<0.01	<0.01
Time to operation (h)	100.8±55.2 96 (24–480)	108.0±55.2 96 (24–408)	100.8±74.4 96 (24–816)	0.08	<0.01
Operation time (mins)	94.1±35.9	93.0±37.0	91.7±35.0	0.40	0.28
Blood loss (ml)	247.0±163.1 200 (10–1600)	236.8±142.2 200 (10–1500)	237.5±149.5 200 (30–1200)	0.32	0.70
Infusion (ml)	1572.3±386.5 1600 (600–3200)	1528.5±352.8 1600 (400–3200)	1558.9±405.1 1600 (400–3700)	0.08	0.12
Stay in hospital (d)	8.5±3.3 7 (4–37)	8.6±2.9 8 (4–37)	9.0±3.8 8 (4–27)	0.01	0.02
Follow-up (m)	41.9±18.7 40.8 (0.0–83.4)	39.4±19.3 39.10 (0.0–83.5)	37.4±19.0 37.29 (0.0–83.9)	<0.01	<0.01
Deaths in hospital	16 (1.6%)	13 (2.0%)	7 (1.0%)	0.31	–
One-year mortality	62 (6.2%)	72 (11.0%)	82 (11.5%)	<0.01	–

Mean+SD/N(%).

^a^ Parametric tests.

^b^ For continuous variables, we used the Kruskal–Wallis rank-sum test, and Fisher’s exact probability test for count variables with a theoretical number <10.

All patients were divided into three groups according to their TTA. The proportions of patients with low (≤6 h), middle (>6, ≤24 h), and high (>24 h) waiting times were 995, 654, and 712, respectively, and the corresponding 1-year mortality rates were 62 (6.2%), 72 (11.0%), and 82 (11.5%). In addition, there were 16, 13, and 7 deaths in the hospital in three subgroups. There were 37 postoperative complications, including deep venous thrombosis, no union of fracture, and dislocation of arthroplasty.

### Univariate analysis of the association between variates and 1-year mortality

Based on the univariate analysis (Table 2) according to the criteria of *P*<0.1 and the previously reported factors, we found these confounding factors to adjust: age, CHD, dementia, time to operation, infusion, transfer, and stay in hospital.

**Table 2:** Effects of factors on 1-year mortality measured by univariate analysis.

	Statistics	OR (95% CI)	*P*
Age (y)	79.44±6.71	1.08 (1.06–1.11)	<0.01
Sex			
Male	743 (31.47%)	1	
Female	1618 (68.53%)	0.79 (0.59–1.06)	0.12
Injury mechanism			
Low velocity trauma	2286 (96.82%)	1	
High velocity trauma	61 (2.58%)	0.51 (0.16–1.65)	0.26
Unclear	14 (0.59%)	2.70 (0.75–9.74)	0.13
Fracture classification			
Intertrochanteric fracture	1745 (73.91%)	1	
Femoral neck fracture	616 (26.09%)	0.91 (0.66–1.26)	0.59
Hypertension	1154 (48.88%)	1.16 (0.88–1.54)	0.29
Diabetes	459 (19.44%)	1.24 (0.89–1.74)	0.21
CHD	1215 (51.46%)	1.33 (1.00–1.76)	0.05
Arrhythmia	757 (32.06%)	1.12 (0.83–1.50)	0.47
Hemorrhagic stroke	48 (2.03%)	1.43 (0.60–3.40)	0.42
Ischemic stroke	665 (28.17%)	1.19 (0.88–1.61)	0.26
Cancer	65 (2.75%)	1.84 (0.93–3.67)	0.08
Multiple injuries	163 (6.90%)	0.78 (0.42–1.42)	0.41
Dementia	89 (3.77%)	2.27 (1.30–3.98)	<0.01
COPD	137 (5.80%)	1.44 (0.85–2.45)	0.17
Hepatitis	70 (2.96%)	1.49 (0.73–3.03)	0.28
Gastritis	42 (1.78%)	0.76 (0.23–2.48)	0.65
aCCI	4.2±1.1	1.52 (1.34–1.73)	<0.01
Treatment strategy			
CRIF/ORIF	1724 (73.02%)	1	
HA	602 (25.50%)	1.01 (0.73–1.39)	0.95
THA	35 (1.48%)	0.29 (0.04–2.12)	0.22
TTA (h)	79.15±237.19	1.00 (1.00–1.00)	0.03
Time to operation (h)	102.96±61.68	1.05 (1.00–1.10)	0.04
Blood loss (ml)	241.32±153.49	1.00 (1.00–1.00)	0.50
Operation time (mins)	93.07±35.95	1.00 (1.00–1.00)	0.82
Infusion (ml)	1556.04±383.40	1.00 (1.00–1.00)	0.06
Stay in hospital (d)	8.69±3.38	1.04 (1.01–1.08)	0.02
Transferred			
No	1124 (48.08%)	1.0	
Yes	1214 (51.92%)	1.40 (1.06–1.87)	0.02
Geography			
Suburban	1704 (72.88%)	1.0	
Urban	634 (27.12%)	0.89 (0.65–1.23)	0.49
Insurance status			
Residents medical insurance	1357 (58.04%)	1.0	
Employee medical insurance	981 (41.96%)	0.91 (0.69–1.22)	0.54
Timing of injuries			
Daytime	1870 (79.98%)	1.0	
Night-time	468 (20.02%)	0.94 (0.66–1.34)	0.72

### Multivariate analysis between preoperative TTA and 1-year mortality

We used a binary logistic regression model to assess the correlation between TTA and 1-year mortality incidence. In Table 3, TTA was not associated with 1-year mortality (OR=1.00; 95% CI: 1.00–1.00; *P*=0.09) in the adjusted model. When we divided TTA into different subgroups, the results were unstable. In the fully-adjusted model, compared to the low (≤6 h) TTA subgroup, the middle (>6, ≤24 h) TTA subgroups could increase the 1-year mortality 1.70-fold (OR=1.70; 95% CI: 1.18–2.46; *P*<0.01), the high (>24 h) TTA subgroups could increase the 1-year mortality 1.68-fold (OR=1.68; 95% CI: 1.11–2.54; *P*<0.01). However, the interval between high/middle was 1.68/1.70=0.98, which is less than 1.68 or 1.70.

**Table 3:** Univariate and multivariate results by linear regression.

Exposure	Crude model	Adjusted model
TTA (h)	1.00 (1.00–1.00) 0.03	1.00 (1.00–1.00) 0.09
TTA (h) tertiles		
≤6	1.0	1.0
>6, ≤24	1.87 (1.30–2.66) **<**0.01	1.70 (1.18–2.46) **<**0.01
>24	1.96 (1.39–2.77) **<**0.01	1.68 (1.11–2.54) **<**0.01
*P* for trend	**<**0.01	**<**0.01

Data in Table: OR (95% CI) *P*-value.

Outcome variable: one-year mortality.

Exposed variables: TTA.

Adjusted model was adjusted for: age, CHD, dementia, time to operation, infusion, transferred and stay in hospital.

### Curve fitting and analysis of threshold or saturation effect

**Figure 2:**
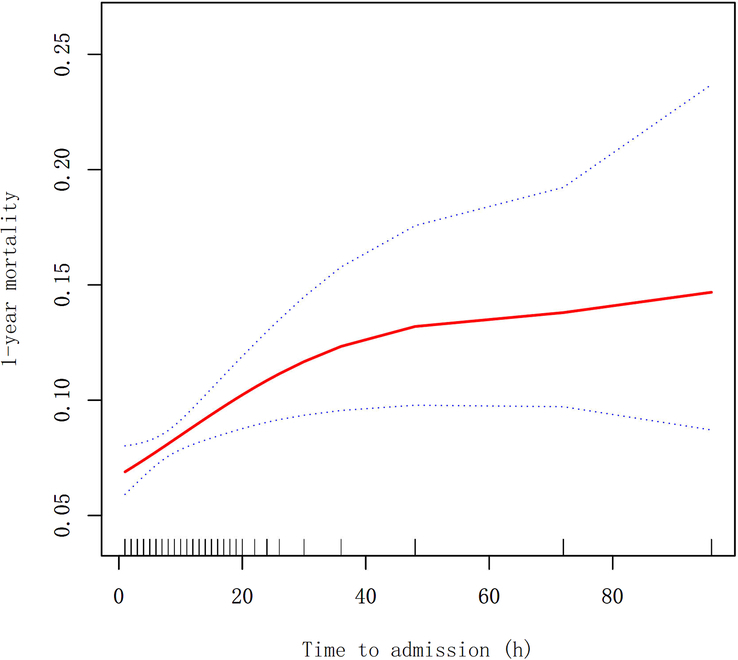
Curve fitting between time to admission and 1-year mortality. They were adjusted for age, CHD, dementia, time to operation, infusion, transferred, and stay in hospital. The red solid line means fitting line and the dashed line means 95% CI.

As shown in Figure 2, there was a curvilinear correlation between TTA and 1-year mortality after adjusting for confounding factors. Through two-piecewise logistic regression analysis, we found that 9 h was an inflection point, as shown in Table 4. When TTA was less than 9 h, there was a correlation between TTA and 1-year mortality of patients. For every 1-hour increase in TTA, the 1-year mortality of patients increased by 9% (OR=1.09, 95% CI: 1.03–1.16; *P*<0.01). When TTA was greater than 9 h, the 1-year mortality rate of patients became relatively stable and no longer changed with TTA (OR=1.00, 95% CI: 1.00–1.00; *P*=0.26).

**Table 4:** Nonlinearity of preoperative TTA and 1-year mortality.

Outcome:	OR (95% CI) *P*
Fitting model by stand linear regression	1.00 (1.00–1.00) 0.09
Fitting model by two-piecewise linear regression	
Inflection point	9 h
<9 h	1.09 (1.03–1.16) **<**0.01
>9 h	1.00 (1.00–1.00) 0.26
*P* for log-likelihood ratio test	**<**0.01

Outcome variable: one-year mortality.

Exposure variables: Time to admission (h).

Adjusted for age, CHD, dementia, time to operation, infusion, transferred and stay in hospital.

### The Kaplan–Meier survival curves

**Figure 3:**
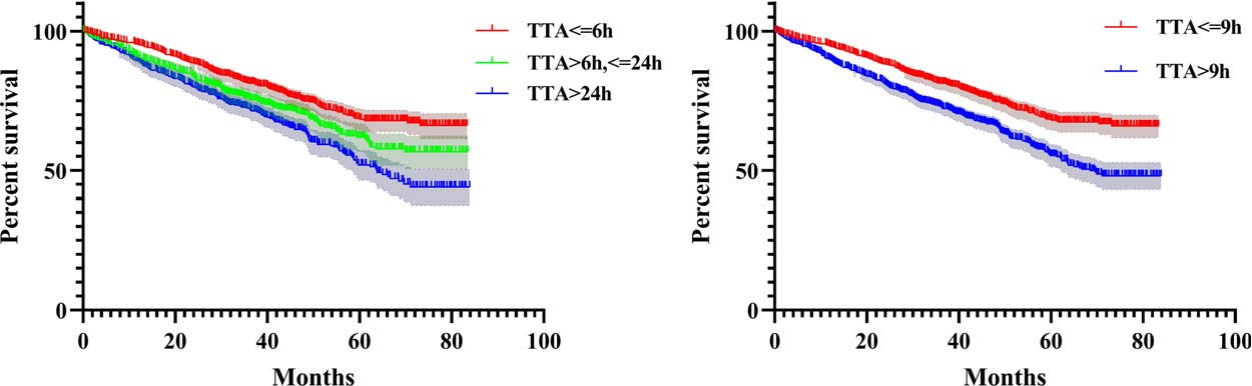
The Kaplan–Meier survival curve according to time to admission subgroups and inflection point of 9 h.

The Kaplan–Meier survival curves according to TTA subgroups and inflection point of 9 h are shown in Figure 3.

### Stratification analysis

We performed a stratification analysis to test the stability of the nonlinearity association, as shown in Table 5. When the patients were divided into subgroups as age, sex, aCCI, transferred, CHD, dementia, time to operation, infusion, and stay in hospital, we found that even though there were different inflection points, most of *P* for log-likelihood ratio test reached the statistical differences.

**Table 5:** Stratification analysis.

Subgroups	No. of patients	TTA (h)	Inflection point (h)	OR (95% CI) *P*-value <Inflection point	OR (95% CI) *P*-value >Inflection point	*P* for log-likelihood ratio test
Age						
65<age≤75	667	67.1±153.2	288	1.00 (1.00–1.01) 0.0593	0.99 (0.98–1.01) 0.3913	0.064
75<age≤85	1249	85.9±294.0	2	2807408.17 (0.00–Inf) 0.9822	1.00 (1.00–1.00) 0.1267	0.036
85<age	445	78.4±143.4	336	1.00 (0.99–1.00) 0.2202	1.00 (1.00–1.01) 0.0384	0.047
Sex						
Female	1618	79.6±246.8	30	1.04 (1.01–1.06) 0.0048	1.00 (1.00–1.00) 0.6242	0.004
Male	743	78.2±215.0	2	2659279.31 (0.00–Inf) 0.9792	1.00 (1.00–1.00) 0.2129	0.017
aCCI						
≤4	1569	67.6±209.9	10	1.10 (1.03–1.18) 0.0061	1.00 (1.00–1.00) 0.4246	0.005
>4	792	102.1±282.3	2	1760342.48 (0.00–Inf) 0.9801	1.00 (1.00–1.00) 0.3862	0.06
Transferred						
Yes	1214	138.6±301.7	24	1.01 (0.99–1.04) 0.2113	1.00 (1.00–1.00) 0.1990	0.217
No	1124	12.0±18.9	17	1.07 (1.03–1.12) 0.0007	0.98 (0.95–1.00) 0.0913	<0.001
CHD						
Yes	1215	78.4±203.3	8	1.12 (1.02–1.24) 0.0178	1.00 (1.00–1.00) 0.0844	0.041
No	1146	79.9±268.6	2	2554552.96 (0.00–Inf) 0.9818	1.00 (1.00–1.00) 0.9171	0.015
Dementia						
Yes	89	107.8±159.2	4	482968.39 (0.00–Inf) 0.9942	1.00 (1.00–1.00) 0.5730	0.267
No	2272	78.0±239.7	9	1.10 (1.03–1.17) 0.0025	1.00 (1.00–1.00) 0.3303	0.002
Time to operation (d)						
≤2	558	125.0±354.7	24	1.02 (0.98–1.06) 0.2898	1.00 (1.00–1.00) 0.1347	0.298
>2	1803	65.0±184.6	8	1.14 (1.05–1.23) 0.0020	1.00 (1.00–1.00) 0.8464	0.001
Infusion (ml)						
≤1600	1779	72.7±203.7	17	1.03 (1.00–1.06) 0.0358	1.00 (1.00–1.00) 0.1686	0.038
>1600	582	95.3±298.7	7	1.37 (1.10–1.71) 0.0043	1.00 (1.00–1.00) 0.5807	0.002
Stay in hospital (d)						
≤7	858	81.9±298.3	11	1.07 (0.98–1.16) 0.1509	1.00 (1.00–1.00) 0.2728	0.146
>7	1503	77.6±194.0	9	1.09 (1.02–1.17) 0.0165	1.00 (1.00–1.00) 0.5486	0.015

Inf, infinity.

## Discussion

The time from fracture to operation can be divided into two subcomponents: the time from fracture to admission and the time from admission to operation. Most previous studies only explored the correlation between postoperative mortality and preoperative delay in time from admission to operation, ignoring the influence of TTA^11,12^. This study found that the delay in TTA was a probable risk factor for 1-year mortality, consistent with previous literature results^15^. Interestingly, this study shows a curve relationship between TTA and the 1-year mortality rate after operation for elderly patients with hip fractures. We found that 9 h was an inflection point in the curve relationship. When TTA was less than 9 h, the 1-year mortality of patients increased by 9% (OR=1.09) for every 1 h increase in TTA delay. When TTA was greater than 9 h, the 1-year mortality of patients tended to be stable and no longer increased (OR=1.00). Therefore, it is necessary to diagnose hip fractures and make an admission as early as possible.

The 1-year mortality of patients after hip surgery is estimated to be about 30%^16^, the mortality was mainly related to postoperative complications, such as comorbidity^17^, age^18^, sex, poor preoperative walking status^19^, weakness^20^, infection^21^, anesthesia techniques^22^, anesthesia class^19^, time from fracture to operation^23^, time from admission to operation^11,23–26^, and time from fracture to admission^25,27^, according to previous studies.

Many developed countries’ studies defaulted to the delay from admission to surgery as the preoperative delay time^12^ because they believed patients could be quickly sent to the appropriate hospital for treatment. However, this is challenging to achieve in developing countries like China. The imbalance of medical resources and the need for surgeons make the time from admission to surgery longer^28^. At the same time, many hip fracture patients are older and often suffer from underlying diseases, such as diabetes, hypertension, lung disease, etc. Hypertension, diabetes, and other comorbidities are considered the leading causes of delayed hip fracture surgery, and the more serious the comorbidity, the longer the preoperative waiting time, according to the study of Pincus *et al*.^29^, thus affecting the prognosis. In our study, 1154 (48.88%) patients had hypertension and 459 (19.44%) had diabetes. In additional univariate linear regression, hypertension (β=0.32; 95% CI: 0.11–0.53; *P*<0.001) was associated with the time to operation, but not diabetes (β=0.19; 95% CI: −0.07–0.46; *P*=0.15). The comorbidity was an essential reason for surgical delay.

Recently, more and more studies have found that delayed TTA is a probable risk factor that is easily overlooked and has important implications for the prognosis of patients, especially in developing countries^25–27^. There was some evidence about the relationships between TTA and postoperative mortality. A prospective cohort study of 78 patients, 3.45 days delay in injury-to-admission, showed that TTA was a risk factor in 30-day mortality^30^. In a retrospective study, He *et al*.^25^ found that admission delay of more than 1 week significantly increased 1-year mortality by 76%. In a 3893 hip fractures study, the delay in TTA by indirect admissions via hospital transfer would increase the perioperative morbidity and mortality rates^31^. In our study, hospital transfer was associated with increasing postoperative mortality (OR=1.40; 95% CI: 1.06–1.87; *P*=0.02), which is similar to the previous study^31^. In addition, Vidal *et al*.^32^ proved that increased TTA was associated with reduced survival to hospital discharge by 9% and reduced survival at 1-year after surgery by 7%. Different reasons can cause delays in TTA. First, in developing countries, hip fractures are rarely seen as a condition requiring emergency surgery due to low health literacy and lack of relevant medical knowledge in some areas. Patients and family members underestimate the injury’s severity and the disease’s prognosis, leading to a higher incidence of delayed admissions than in developed countries. Secondly, equal access to appropriate surgical treatment is also essential to the admission delay. Some patients may need to be transferred to another hospital after admission because the hospital lacks orthopedic surgery capabilities. These can be optimized by raising social awareness and adjusting health policies.

It may be expected that a delay in hospitalization may affect the first phases of the clinical course. However, there was no difference in hospital mortality among the three groups. Two main reasons could explain this result. First, this study is an observational cohort study; it could not provide the causal connection, or the logistical relationship that delays in TTA could increase mortality. Second, we often wait for preoperative examination to rule out contraindications to surgery and invite internal medicine to assist with treatment, if necessary, in our trauma center. Even though this process would lead to a delay in time from admission to operation, and the overall mortality in hospital is only 1.5% (36/2361) in this study, less than 3% from another cohort study of 2464 consecutive patients >65 years in America^33^. Therefore, it is difficult to find the difference in mortality among the three groups under a low percentage.

Several factors were significantly more frequent in the TTA >24 h group, related to higher 1-year mortality. Compared to the groups of TTA ≤6 h and 6 h<TTA≤24 h, there were more patients with arrhythmia, hemorrhagic stroke, ischemic stroke, and dementia in this study, as shown in Table 1. It was reported that perioperative atrial fibrillation was associated with increased 1-year mortality in elderly patients after repair of hip fracture by 6.7-fold^34^. Hjelholt *et al*.^35^ observed excess short-term mortality in patients with stroke and hip fracture, and the clinicians were encouraged to pay rigorous attention to early complications among hip fracture patients with stroke. Also, a meta-analysis of 30 studies revealed that there were significant negative impacts of dementia on mortality at different periods^36^. Therefore, we hypothesized that these pathophysiological mechanisms lead to higher mortality in admission delays.

As for the total time from injury to operation, 23.57% of patients received the operation in 2 days, 44.1% in 3 days, and 65.0% in 4 days in this study. Thus, most patients received the operation in 4 days. Because we often wait for preoperative examination to rule out contraindications and invite internal medicine to assist in our hospital. Even though this leads to a delay in time to operation, the 1-year mortality was 9.2% (216/2361), less than the 33% in a study from other regions^37^.

As far as we know, delays in TTA are unavoidable in developed countries. In their study of Canadian hip fractures, Vidal *et al*.^38^ found that 10% of patients were hospitalized at least 2 days after the fracture. At the same time, the uneven distribution of population and medical resources prolongs the TTA, resulting in poor prognosis of patients^39^. Delayed admission for over 1 week was significantly associated with higher 1-year mortality, according to He *et al*.^25^ study containing 970 patients. Li *et al*.^15^ found that surgical delay, especially from injury to hospitalization, is an essential factor affecting the early mortality of hip fractures. According to their study, the survival rate of patients with an interval of more than 2 days from injury to hospitalization was lower than that of patients with an interval of less than or equal to 2 days from injury to hospitalization. Our study found that when TTA was less than 9 h, there was a correlation between TTA and 1-year mortality of patients. For every 1 h increase in TTA, the 1-year mortality of patients increased by 9%. When TTA was greater than 9 h, the mortality rate tended to be stable and maintained at a high level. This will allow us to obtain more details and preventive measures to guide the treatment of hip fractures in older people, especially in the upcoming global population aging challenge.

The long-term delay between fracture and hospitalization mainly reflects the need for more relevant medical knowledge and treatment principles of hip fracture in patients or patients’ nursing staff. Corresponding policies should be formulated to popularize health knowledge. It is necessary to help primary medical institutions and patients understand the severity of hip fractures and send patients to qualified hospitals in time to reduce surgical delays. This will not only optimize the prognosis and survival rate of elderly patients with hip fracture surgery but also help to reduce postoperative complications, stay in hospital, and costs^27^.

This study has the following significant advantages: First, due to the large sample size, we recruited 2361 patients who met the criteria. Secondly, we adjusted various factors and analyzed the linear and curve relationships. Through multiple methods, we determined the reliability of the result. However, this study still has some limitations. Firstly, this study is observational and single-center, and the causal relationship between TTA and prognosis has yet to be established. This will need to be further confirmed in future studies. Secondly, our study population was from Northwest China, with geographical and ethnic limitations. This study’s findings should be applied cautiously to populations from other regions.

## Conclusion

TTA is a probable predictor of 1-year mortality. We found that 9 h was an inflection point. If TTA is less than 9 h, the 1-year mortality increases with TTA. If TTA exceeds 9 h, the mortality rate tends to be stable and maintained at a high level. Therefore, older adults suspected of having a hip fracture should be admitted to the hospital as early as possible, thereby reducing patient mortality.

## Ethical approval

The study was approved by the Ethics Committee of the Honghui Hospital, Xi’an Jiaotong University (No. 202201009). All human-related procedures followed the 1964 Declaration of Helsinki and its later amendments. The study has been reported according to the STROCSS 2021 guidelines.

## Consent

Consent for publication: Not applicable.

Consent to Publish: The work described has not been published before (except in the form of an abstract or as part of a published lecture, review, or thesis); it is not under consideration for publication elsewhere, and all co-authors have approved its publication.

## Source of funding

This work was supported by the Foundation of Xi’an Municipal Health Commission (Grant Number: 2024ms15).

## Author contribution

According to the definition given by the International Committee of Medical Journal Editors (ICMJE), the authors listed above qualify for authorship based on making one or more of the substantial contributions to the intellectual content of the following: B.-F.Z. and P.X.: conceived and designed the study; B.-F.Z., S.-L.X., and Z.Y.: performed the study; B.-F.Z.: analyzed the data; B.-F.Z. and S.-L.X.: wrote the manuscript.

## Conflicts of interest disclosure

The authors declare that they have no competing interests.

## Research registration unique identifying number (UIN)

This study is registered on the website of the Chinese Clinical Trial Registry (ChiCTR: ChiCTR2200057323).

## Guarantor

Bin-Fei Zhang, e-mail: zhangbf07@gmail.com.

## Data availability statement

Xi’an Honghui Hospital implemented the data. According to relevant regulations, the data could not be shared but could be requested from the corresponding author.

## References

[1] Gong X Li X Zhang L. Current status and distribution of hip fractures among older adults in China. Osteoporos Int 2021;32:1785–1793. 33655399 10.1007/s00198-021-05849-y

[2] Cooper C Cole Z Holroyd C. Secular trends in the incidence of hip and other osteoporotic fractures. Osteoporos Int 2011;22:1277–1288. 21461721 10.1007/s00198-011-1601-6 PMC3546313

[3] Xia W He S Xu L. Rapidly increasing rates of hip fracture in Beijing, China. J Bone Miner Res 2012;27:125–129. 21956596 10.1002/jbmr.519

[4] Kannus P Parkkari J Sievänen H. Epidemiology of hip fractures. Bone 1996;18:57S–63S. 8717549 10.1016/8756-3282(95)00381-9

[5] Haentjens P Magaziner J Colón-Emeric C. Meta-analysis: excess mortality after hip fracture among older women and men. Ann Intern Med 2010;152:380–390. 20231569 10.1059/0003-4819-152-6-201003160-00008 PMC3010729

[6] Simunovic N Devereaux P Sprague S. Effect of early surgery after hip fracture on mortality and complications: systematic review and meta-analysis. CMAJ 2010;182:1609–1616. 20837683 10.1503/cmaj.092220 PMC2952007

[7] Kjaervik C Gjertsen J Engeseter L. Waiting time for hip fracture surgery: hospital variation, causes, and effects on postoperative mortality : data on 37,708 operations reported to the Norwegian Hip fracture Register from 2014 to 2018. Bone Jt Open 2021;2:710–720. 34472378 10.1302/2633-1462.29.BJO-2021-0079.R1 PMC8479844

[8] Greve K Modig K Talbäck M. No association between waiting time to surgery and mortality for healthier patients with hip fracture: a nationwide Swedish cohort of 59,675 patients. Acta Orthop 2020;91:396–400. 32326789 10.1080/17453674.2020.1754645 PMC8023952

[9] Walter N Szymski D Kurtz S. Epidemiology and treatment of proximal femoral fractures in the elderly U.S. population. Sci Rep 2023;13:12734. 37543668 10.1038/s41598-023-40087-8 PMC10404231

[10] Mak J Cameron I March L. Evidence-based guidelines for the management of hip fractures in older persons: an update. Med J Aust 2010;192:37–41. 20047547 10.5694/j.1326-5377.2010.tb03400.x

[11] Maheshwari K Planchard J You J. Early surgery confers 1-year mortality benefit in hip-fracture patients. J Orthop Trauma 2018;32:105–110. 29065037 10.1097/BOT.0000000000001043

[12] Uzoigwe C Burnand H Cheesman C. Early and ultra-early surgery in hip fracture patients improves survival. Injury 2013;44:726–729. 23010072 10.1016/j.injury.2012.08.025

[13] Mathew G Agha R S. Group. STROCSS 2021: strengthening the reporting of cohort, cross-sectional and case-control studies in surgery. Int J Surg 2021;96:106165. 34774726 10.1016/j.ijsu.2021.106165

[14] Guman D Sijia C Weiwei L. Risk factors for patellar clunk or crepitation after primary total knee arthroplasty: a systematic review and meta-analysis. J Knee Surg 2021;34:1098–1099. 32131098 10.1055/s-0040-1701515

[15] Li Y Lin J Wang P. Effect of time factors on the mortality in brittle hip fracture. J Orthop Surg Res 2014;9:37. 24884818 10.1186/1749-799X-9-37 PMC4025195

[16] Sing C Lin T Bartholomew S. Global epidemiology of hip fractures: secular trends in incidence rate, post-fracture treatment, and all-cause mortality. J Bone Miner Res 2023;38:1064–1075. 37118993 10.1002/jbmr.4821

[17] Xing F Luo R Chen W. The risk-adjusted Charlson comorbidity index as a new predictor of one-year mortality rate in elderly Chinese patients who underwent hip fracture surgery. Orthop Traumatol Surg Res 2021;107:102860. 33609760 10.1016/j.otsr.2021.102860

[18] Espinosa K Gélvez A Torres L. Pre-operative factors associated with increased mortality in elderly patients with a hip fracture: a cohort study in a developing country. Injury 2018;49:1162–1168. 29674111 10.1016/j.injury.2018.04.007

[19] Hu F Jiang C Shen J. Preoperative predictors for mortality following hip fracture surgery: a systematic review and meta-analysis. Injury 2012;43:676–685. 21683355 10.1016/j.injury.2011.05.017

[20] Yan B Sun W Wang W. Prognostic significance of frailty in older patients with hip fracture: a systematic review and meta-analysis. Int Orthop 2022;46:2939–2952. 36227383 10.1007/s00264-022-05605-9

[21] Chen B Liu Y Chen C. Correlation between C-reactive protein and postoperative mortality in patients undergoing hip fracture surgery: a meta-analysis. J Orthop Surg Res 2023;18:182. 36894998 10.1186/s13018-023-03516-y PMC9996565

[22] Cao M Zhang Y Sheng R. General anesthesia versus regional anesthesia in the elderly patients undergoing hip fracture surgeries: a systematic review and meta-analysis of randomized clinical trials. World J Surg 2023;47:1444–1456. 36826487 10.1007/s00268-023-06949-y

[23] Leer-Salvesen S Engesæter L Dybvik E. Does time from fracture to surgery affect mortality and intraoperative medical complications for hip fracture patients? An observational study of 73 557 patients reported to the Norwegian Hip Fracture Register). Bone Joint J 2019;9:1129–1137. 10.1302/0301-620X.101B9.BJJ-2019-0295.R1 31474142

[24] Nyholm A Gromov K Palm H. Time to surgery is associated with thirty-day and ninety-day mortality after proximal femoral fracture: a retrospective observational study on prospectively collected data from the Danish Fracture Database Collaborators. J Bone Joint Surg Am 2015;97:1333–1339. 26290084 10.2106/JBJS.O.00029

[25] He W You Y Sun K. Admission delay is associated with worse surgical outcomes for elderly hip fracture patients: a retrospective observational study. World J Emerg Med 2020;11:27–32. 31893000 10.5847/wjem.j.1920-8642.2020.01.004 PMC6885583

[26] Paul P Issac RJ. Delay in time from fracture to surgery: a potential risk factor for in-hospital mortality in elderly patients with hip fractures. J Orthop 2018;15:375–378. 29881157 10.1016/j.jor.2018.03.001 PMC5990208

[27] ChenXLiaoZShenY. The relationship between pre-admission waiting time and the surgical outcomes after hip fracture operation in the elderly. J Nutr Health Aging 2021;25:951–955.34545913 10.1007/s12603-021-1656-9

[28] Pincus D Wasserstein D Ravi B. Reporting and evaluating wait times for urgent hip fracture surgery in Ontario, Canada. CMAJ 2018;190:E702–E709. 29891474 10.1503/cmaj.170830 PMC5995591

[29] Pincus D Ravi B Wasserstein D. Association between wait time and 30-day mortality in adults undergoing hip fracture surgery. JAMA 2017;318:1994–2003. 29183076 10.1001/jama.2017.17606 PMC5820694

[30] Ghosh A Patel S Chouhan D. Pre-hospital delays represent unnoticed intervals that affect mortality rates in geriatric hip fractures: a prospective cohort study. Cureus 2023;15:e44773. 37809112 10.7759/cureus.44773 PMC10557467

[31] Hughes A Brent L Biesma R. The effect of indirect admission via hospital transfer on hip fracture patients in Ireland. Ir J Med Sci 2019;188:517–524. 29974324 10.1007/s11845-018-1854-6

[32] Vidal E Moreira-Filho D Pinheiro R. Delay from fracture to hospital admission: a new risk factor for hip fracture mortality? Osteoporos Int 2012;23:2847–2853. 22297734 10.1007/s00198-012-1917-x

[33] Groff H Kheir M George J. Causes of in-hospital mortality after hip fractures in the elderly. Hip Int 2020;30:204–209. 30909746 10.1177/1120700019835160

[34] Leibowitz D Abitbol C Alcalai R. Perioperative atrial fibrillation is associated with increased one-year mortality in elderly patients after repair of hip fracture. Int J Cardiol 2017;227:58–60. 27846465 10.1016/j.ijcard.2016.11.067

[35] Hjelholt T Johnsen S Brynningsen P. The interaction effect between previous stroke and hip fracture on postoperative mortality: a nationwide cohort study. Clin Epidemiol 2022;14:543–553. 35509521 10.2147/CLEP.S361507 PMC9058007

[36] Hou M Zhang Y Chen A. The effects of dementia on the prognosis and mortality of hip fracture surgery: a systematic review and meta-analysis. Aging Clin Exp Res 2021;33:3161–3172. 33913118 10.1007/s40520-021-01864-5

[37] Guzon-Illescas O Perez Fernandez E Crespí Villarias N. Mortality after osteoporotic hip fracture: incidence, trends, and associated factors. J Orthop Surg Res 2019;14:203. 31272470 10.1186/s13018-019-1226-6 PMC6610901

[38] Vidal E Moreira-Filho D Coeli C. Hip fracture in the elderly: does counting time from fracture to surgery or from hospital admission to surgery matter when studying in-hospital mortality? Osteoporos Int 2009;20:723–729. 18839050 10.1007/s00198-008-0757-1

[39] Wang Y Li Y Qin S. The disequilibrium in the distribution of the primary health workforce among eight economic regions and between rural and urban areas in China. Int J Equity Health 2020;19:28. 32102655 10.1186/s12939-020-1139-3 PMC7045560

